# Preadipocyte factor 1 induces pancreatic ductal cell differentiation into insulin-producing cells

**DOI:** 10.1038/srep23960

**Published:** 2016-04-05

**Authors:** Marie Rhee, Seung-Hwan Lee, Ji-Won Kim, Dong-Sik Ham, Heon-Seok Park, Hae Kyung Yang, Ju-Young Shin, Jae-Hyoung Cho, Young-Bum Kim, Byung-Soo Youn, Hei Sook Sul, Kun-Ho Yoon

**Affiliations:** 1 Division of Endocrinology and Metabolism, Department of Internal Medicine, College of Medicine, The Catholic University of Korea, Seoul, Korea; 2 Convergent Research Consortium for Immunologic Disease, Catholic Research Institutes of Medical Science, The Catholic University of Korea, Seoul, Korea; 3 Division of Endocrinology, Diabetes and Metabolism, Beth Israel Deaconess Medical Center and Harvard Medical School, Boston, MA, USA; 4 Biomedical Research Center, Ulsan University Hospital, University of Ulsan College of Medicine, Ulsan, Korea; 5 Department of Nutritional Science and Toxicology, University of California, Berkeley, CA, USA

## Abstract

The preadipocyte factor 1 (Pref-1) is involved in the proliferation and differentiation of various precursor cells. However, the intracellular signaling pathways that control these processes and the role of Pref-1 in the pancreas remain poorly understood. Here, we showed that Pref-1 induces insulin synthesis and secretion via two independent pathways. The overexpression of Pref-1 activated MAPK signaling, which induced nucleocytoplasmic translocation of FOXO1 and PDX1 and led to the differentiation of human pancreatic ductal cells into β-like cells and an increase in insulin synthesis. Concurrently, Pref-1 activated Akt signaling and facilitated insulin secretion. A proteomics analysis identified the Rab43 GTPase-activating protein as a downstream target of Akt. A serial activation of both proteins induced various granular protein syntheses which led to enhanced glucose-stimulated insulin secretion. In a pancreatectomised diabetic animal model, exogenous Pref-1 improved glucose homeostasis by accelerating pancreatic ductal and β-cell regeneration after injury. These data establish a novel role for Pref-1, opening the possibility of applying this molecule to the treatment of diabetes.

The preadipocyte factor 1 [Pref-1; also called Delta-like protein 1 (Dlk1) or fetal antigen 1 (FA1)] is a preadipocyte secreted protein that plays an inhibitory role in adipogenic differentiation^1^,^2^,^3^. It has also been identified as a novel factor that regulates human mesenchymal stem cell differentiation to osteoblasts and adipocytes^4^,^5^,^6^,^7^. Pref-1 knockout mice display growth retardation, skeletal malformation, accelerated adiposity and increased serum lipid metabolites^8^. Conversely, mice that overexpress Pref-1 in adipose tissue show a decrease in adipose tissue mass, reduced expression of adipocyte markers, and a lower level of adipocyte-secreted hormones, including leptin and adiponectin. Because of decreased adipose tissue development, these mice also suffer from hypertriglyceridaemia, impaired glucose tolerance, and lower insulin sensitivity^1^.

Pref-1 is also expressed in the hepatoblasts, oval cell compartment, and amplifying duct cells of a regenerating liver^6^,^9^. Pref-1 is strongly expressed in the fetal liver between embryonic days (E) 10.5 and E16.5, and is useful as a marker of enrichment of highly proliferative hepatoblasts. In addition, Pref-1 expression was detected in oval cells, which are adult hepatic progenitors, in the rat 2-acetylaminofluorene/partial hepatectomy model. These observations suggest that Pref-1 is implicated in the proliferation and/or differentiation of hepatocytes. For these reasons, many studies have proposed that Pref-1 is not only a marker of adult stem cells, but also a regulator that is involved in the proliferation and differentiation of various precursor cells^2^,^6^.

In the case of the pancreas, Pref-1 is present throughout embryonic development until the postnatal stage. Pref-1 levels increase approximately 5-fold at birth, but then rapidly decreases at 4 days after birth^10^. Previously, we demonstrated that Pref-1 is expressed in the small duct cells of the embryonic pancreas and in regenerating foci after partial pancreatectomy in rats^11^ (Supplementary Figure 1a,b). Thus, Pref-1 might be a useful marker of pancreatic protodifferentiated cells. However, it remains unclear whether Pref-1 plays an important role in pancreatic development and regeneration. Furthermore, the role of the Pref-1 signaling pathway has not been elucidated in pancreatic precursor cells. As pancreatic duct cells are considered as possible progenitor cells of β-cells^12^,^13^,^14^,^15^,^16^, the present study aimed to clarify the molecular mechanism of Pref-1 signaling in pancreatic duct cells and to demonstrate the effect of Pref-1 on the differentiation of pancreatic duct cells into β-like cells and insulin secretion.

## Results

### Pref-1 promotes the phosphorylation of ERK1/2 and Akt independently and induces changes in the expression of FOXO1 and PDX1

**Figure 1:**
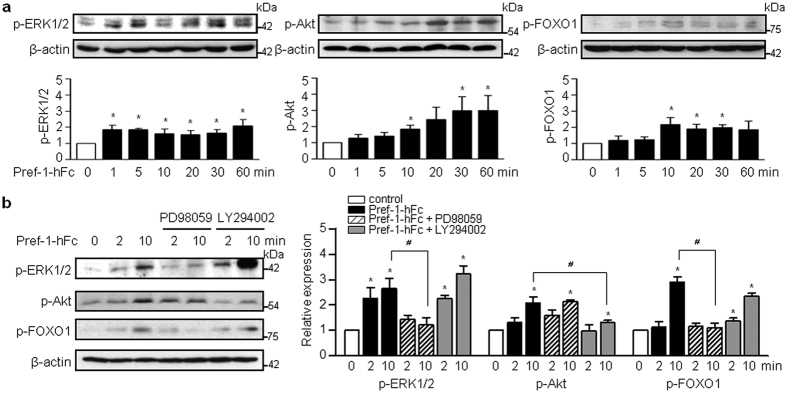
Pref-1 promotes the phosphorylation of ERK1/2 and Akt independently. (**a**) Immunoblot analyses of the phosphorylated forms of ERK1/2, Akt, and FOXO1 in PANC1 cells treated with purified human Pref-1-hFc (50 ng/mL) for the indicated times, and their quantification. n = 3, ^*^P < 0.05 vs 0 min (means ± SE). (**b**) Cells were incubated with 25 μM PD98059 and 20 μM LY294002, which are pharmacological inhibitors of ERK1/2 phosphorylation and Akt phosphorylation, respectively. n = 3, ^*^P < 0.05 vs 0 min, ^*#*^*P* < 0.05 between groups (means ± SE).

Because extracellular signal-regulated kinase (ERK) 1/2 has previously been identified as a downstream target of Pref-1, and forkhead box protein O1 (FOXO1) is directly phosphorylated by ERK and Akt^17^,^18^,^19^, we first investigated the effects of Pref-1 on ERK1/2, FOXO1, and Akt phosphorylation in the PANC1 human pancreas duct cell line. The addition of recombinant human Pref-1-Fc (Pref-1-hFc) first induced the phosphorylation of ERK1/2, followed by the phosphorylation of FOXO1. Akt phosphorylation reached its highest level 30 min after treatment with Pref-1 (Fig. 1a). Overexpression of human Pref-1 vector (pSPORT6-hDLK1) also induced the phosphorylation of ERK1/2, FOXO1 and Akt (Supplementary Figure 2a). To confirm the relationship between ERK1/2, FOXO1, and Akt under the influence of Pref-1, we examined the extent of their phosphorylation after the addition of phosphorylation inhibitors (Fig. 1b). Treatment with PD98059, which is a MAP kinase kinase inhibitor, reduced the phosphorylation of both ERK1/2 and FOXO1, but not that of Akt. Treatment with LY294002, which is a PI3K inhibitor, reduced the phosphorylation of Akt, but not that of ERK1/2 or FOXO1. These results indicate that Pref-1 activates ERK1/2 and Akt independently, and that ERK1/2 signaling precedes FOXO1 phosphorylation.

**Figure 2:**
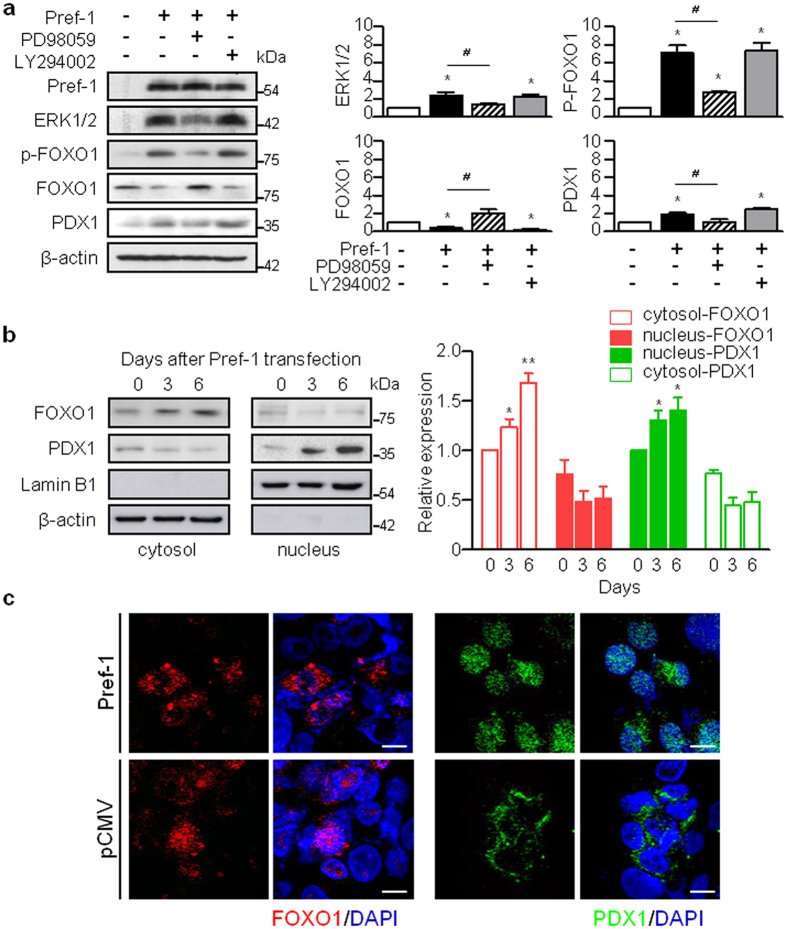
Pref-1 induces nucleocytoplasmic translocation of FOXO1 and PDX1 in PANC1 cells. (**a**) Immunoblot analyses and their quantification of ERK1/2, phosphorylated FOXO1, FOXO1, and PDX1 at day 2 after transfection with either pSPORT6-hDLK1 as Pref-1 or pCMV as control. Cells were treated with 25 μM PD98059 or 20 μM LY294002. n = 3, ^*^P < 0.05 vs pCMV, ^*#*^*P* < 0.05 between groups (means ± SE). (**b**) Protein levels of FOXO1 and PDX1 in the cytoplasmic and nuclear fractions at the indicated times after Pref-1 transfection, and their quantification. n = 3, ^**^P < 0.01 vs day 0 (means ± SE). (**c**) Immunostaining of PDX1 and FOXO1 at day 6 after transfection with either pSPORT6-hDLK1 or pCMV. Scale bar, 5 μm.

Our previous data showed that Pref-1-expressing pancreatic cells coexpress the transcription factor pancreatic duodenal homeobox 1 (PDX1) (Supplementary Figure 1c), which is used as a marker of pancreatic progenitor cells^20^. Therefore, next we examined the effect of Pref-1 on PDX1 expression in PANC1 cells. Pref-1 overexpression decreased the expression of FOXO1 and increased that of ERK1/2, phosphorylated FOXO1 and PDX1 at 2 days after the transfection of the human Pref-1 expression vector, and these changes were blunted by the addition of PD98059 (Fig. 2a). The response to Pref-1 varied according to the cellular compartment; FOXO1 translocation to the cytosol from the nucleus and PDX1 translocation to the nucleus from the cytosol were increased at days 3 and 6 after Pref-1 transfection (Fig. 2b,c). These observations are supported by recent studies suggesting that FOXO1 controls PDX1 by regulating its nucleocytoplasmic translocation^21^,^22^,^23^. This reciprocal translocation of FOXO1 and PDX1 is also regarded as an essential step in the development of pancreatic islet cells. Taken together, these results suggest that Pref-1 induces the ERK-FOXO1-PDX1 pathway and might play a biological role in pancreatic duct cells.

### Pref-1 triggers premature and mature granular protein synthesis via the Akt-Rab43 pathway

**Figure 3:**
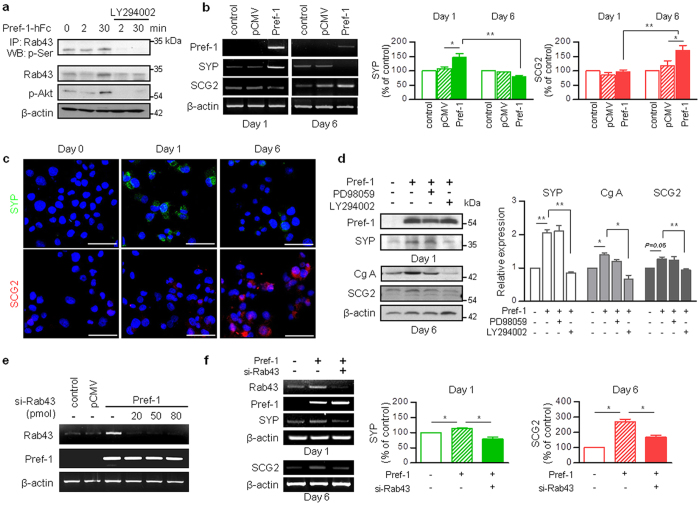
Pref-1 triggers granular protein synthesis via the Akt-Rab43 pathway in PANC1 cells. (**a**) Immunoblot analyses of total and phosphorylated forms of Rab43 in PANC1 cells treated with purified human Pref-1-hFc (50 ng/ml) for the indicated times in the presence or absence of LY294002. n = 3. (**b**) RT-PCR analyses of synaptophysin (SYP) and secretogranin (SCG2) and their quantification at day 1 and 6 after transfection with control, pCMV, or Pref-1. n = 5, ^*^P < 0.05, ^**^P < 0.01 (means ± SE). (**c**) Immunostaining of SYP and SCG2 at days 1 and 6 after transfection. Scale bar, 50 μm. (**d**) Immunoblot analyses and their quantification of SYP, chromogranin A (CgA), and SCG2 in Pref-1-transfected cells in the presence or absence of PD98059 (25 μM) and LY294002 (20 μM). n = 3, ^*^P < 0.05, ^**^P < 0.01 (means ± SE). (**e**) Efficiency of Rab43 knockdown in Pref-1-transfected cells using si-Rab43 at three different concentrations. (**f**) RT-PCR analyses of SYP and SCG2 and their quantification after transfection with Pref-1 and 50 pmol of si-Scr or si-Rab43 at day 1 and 6, respectively. n = 3, ^*^P < 0.05 (means ± SE).

Unlike ERK1/2, Akt showed little relationship with transcription factors after Pref-1 overexpression as evidenced by unchanged expression of ERK1/2, FOXO1 or PDX1 by the addition of LY294002 (Figs 1b and 2a). Consequently, we searched the downstream signaling molecules of Akt phosphorylation. Two-dimensional gel electrophoresis and matrix-assisted laser desorption/ionization time-of-flight (MALDI-TOF) mass spectrometry were used for proteomics analysis. Two days after transfection, cell lysates were prepared from matched samples, using nontransfected cells as the control and Pref-1-transfected cells as the experimental group. The comparison of the matched samples revealed that some proteins were notable because of either their upregulation or downregulation (Supplementary Table 1, Supplementary Figure 3). Among them, we focused on Rab43, which is known to play a role in the intracellular secretory machinery by participating in endoplasmic reticulum to Golgi trafficking^24^. The expression of phosphorylated and total Rab43 was increased after treatment with Pref-1-hFc (Fig. 3a). Treatment with LY294002 inhibited not only the expression of phosphorylated Akt, but also that of phosphorylated Rab43. Based on these findings, we assumed that the overexpression of Pref-1 participates in the upregulation of Rab43 via the Akt pathway.

Pref-1-expressing pancreatic cells coexpress synaptophysin (Supplementary Figure 1d), which is used as a marker of endocrine precursor cells^25^ and is expressed on neurosecretory vesicles^26^. As the possible product of upregulated- and phosphorylated Rab43, we examined the synthesis of granular proteins after Pref-1 overexpression. Synaptophysin formation increased at day 1 after Pref-1 transfection, and the expression of secretogranin (SCG2) increased at day 6 after transfection (Fig. 3b,c). In Pref-1-transfected cells treated with LY294002, but not with PD98059, all granular protein synthesis was suppressed (Fig. 3d). To assess directly whether Pref-1-Akt-dependent Rab43 protein synthesis and phosphorylation contribute to secretory granular protein expression, we treated Pref-1-overexpressing cells with si-Rab43 (Fig. 3e). When cells were treated with 50 pmol si-Rab43, Pref-1-induced synaptophysin and SCG2 synthesis were reduced at day 1 and day 6, respectively (Fig. 3f). These results suggest that Pref-1 overexpression triggers the synthesis of granular proteins, which are normally expressed in pancreatic endocrine cells, and that this was inhibited by the inhibition of Akt or knockdown of Rab43. Taken together, our data confirm that Pref-1 promotes premature and mature granular protein synthesis via the Akt-Rab43 pathway.

### Pref-1 promotes insulin synthesis and glucose-stimulated insulin secretion via the ERK1/2 and Akt pathway independently

**Figure 4:**
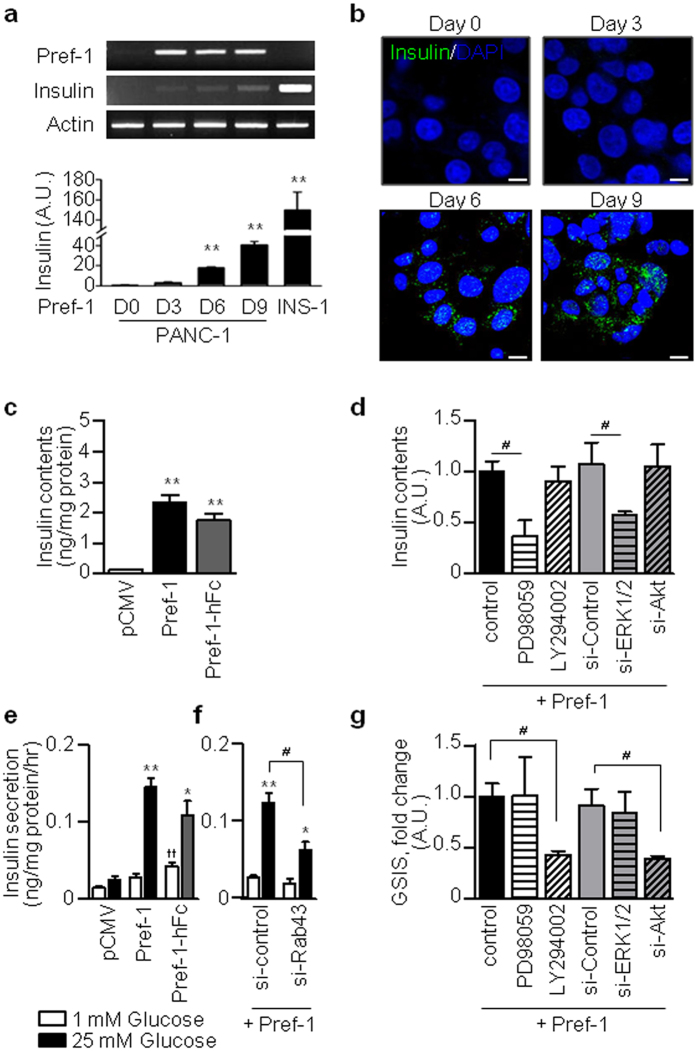
Pref-1 promotes insulin synthesis and glucose-stimulated insulin secretion (GSIS) via the ERK1/2 and Akt pathway independently in PANC1 cells. (**a,b**) RT-PCR analyses and immunostaining of insulin in PANC1 cells transfected with Pref-1 at the indicated times. INS-1 cells are indicated as a positive control. n = 3, ^**^P < 0.01 vs D0 (means ± SE). Scale bar, 5 μm. (**c**) Cellular insulin content in Pref-1-overexpressing cells and human Pref-1-Fc treated cells at day 9 after treatments. n = 5, ^**^P < 0.01 vs pCMV (means ± SE). (**d**) Cellular insulin content in Pref-1-overexpressing cells at day 9 with inhibition of ERK pathway (PD98059 or si-ERK1/2) or Akt pathway (LY294002 or siAkt). n = 5 ~ 6, ^#^*P* < 0.05 between groups (means ± SE). (**e**) GSIS in Pref-1-overexpressing cells and human Pref-1-Fc treated cells at day 9. n = 5, ^*^P < 0.05, ^**^P < 0.01 vs 1 mM glucose, ^††^*P* < 0.01 vs pCMV (means ± SE). (**f**) GSIS in Pref-1-overexpressing cells at day 9 with si-Rab43 treatment. n = 6, ^*^P < 0.05, ^**^P < 0.01 vs 1 mM glucose, ^#^*P* < 0.05 between groups (means ± SE). (**g**) GSIS in Pref-1-overexpressing cells at day 9 with inhibition of ERK pathway (PD98059 or si-ERK1/2) or Akt pathway (LY294002 or siAkt). Fold changes in GSIS was expressed as the insulin secretion ratio at 25 mM glucose compared to 1 mM glucose. n = 5 ~ 6, ^#^*P* < 0.05 between groups (means ± SE).

Next, we examined how insulin synthesis and secretion, the specific phenotypes of mature pancreatic β-cells, are affected by the overexpression of Pref-1 in pancreatic duct cells. Pref-1 transfection increased the expression of the insulin mRNA and the formation of insulin-positive cells (Fig. 4a,b). The expression level of insulin in INS-1 cells was approximately fourfold higher than that in PANC1 cells. To evaluate the differences in insulin quantity, we compared both the insulin-to-cell and insulin-to-culture medium ratios. Intracellular insulin content and glucose-stimulated insulin secretion (GSIS) was significantly increased in Pref-1-treated and Pref-1-transfected cells (Fig. 4c,e). Similarly, intracellular C-peptide content and glucose-stimulated C-peptide secretion was significantly increased in Pref-1-transfected cells (Supplementary Figure 4). Interestingly, the Pref-1-induced increase in insulin content was decreased by PD98059 or si-ERK1/2, but not by LY294002 or si-Akt (Fig. 4d), whereas GSIS was blunted by inhibition of Akt and si-Rab43 (Fig. 4f,g). This increase in GSIS by Pref-1 was not observed in isolated rat islets, indicating that Pref-1 does not have the same role in mature β-cells (Supplementary Figure 5). These data suggest that the Pref-1-induced increase in cellular insulin content was controlled by the ERK pathway, whereas physiological insulin release was controlled by the Akt-Rab43 pathway (Fig. 6).

### Pref-1 regulates pancreas regeneration after pancreatic injury and improves glucose homeostasis

**Figure 5:**
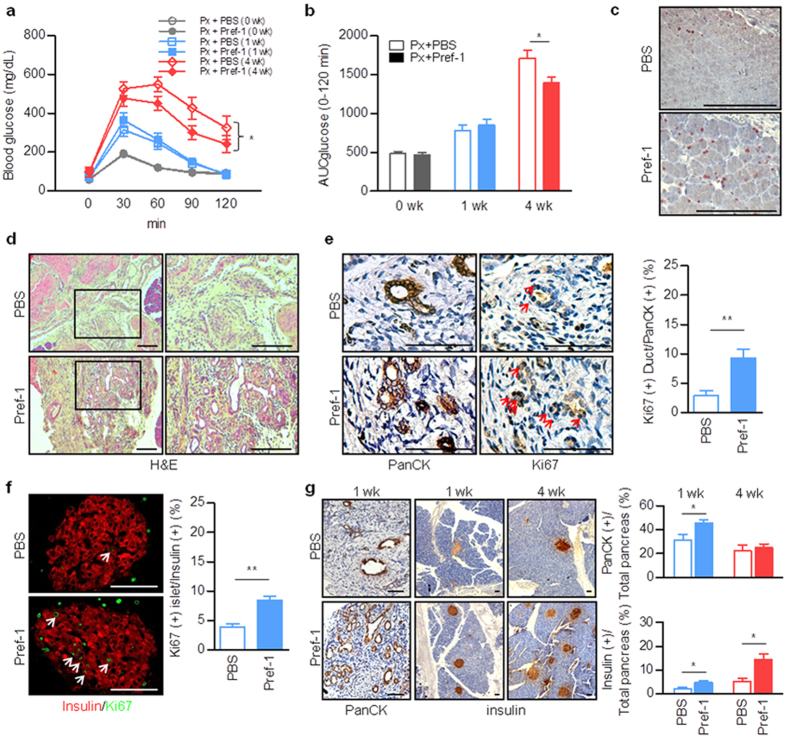
Pref-1 regulates pancreas regeneration and improves glucose homeostasis. (**a,b**) Intraperitoneal glucose tolerance test (**a**) and the area under the curve (AUC) for glucose (**b**) in pancreatectomised (Px) rats treated with mouse Pref-1-Fc (4 μg/kg) or PBS. n = 25 (0 wk), 18 ~ 19 (1 wk), and 7 ~ 8 (4 wk) per group. ^*^P < 0.05 (means ± SE). (**c**) Immunostaining of Ki67 in pancreas at 1 week after pancreatectomy. Scale bar, 100 μm. (**d**) H&E staining of the pancreas at 1 wk after pancreatectomy. Scale bar, 100 μm. (**e–g**) Representative images and quantification of the ratios of Ki67-positive ducts (**e**) and islets (**f**) and the mean areas of duct or insulin at 1 wk and 4 wk after pancreatectomy (**g**) using the positive cell or pixel count algorithm on the digital images captured. n = 8 ~ 9, ^*^P < 0.05, ^**^P < 0.01 (means ± SE). Scale bar, 50 μm (**e,f**), 100 μm (**g**).

Pancreatectomised rats were treated with recombinant mouse Pref-1-Fc (Pref-1-mFc) via daily intraperitoneal (IP) injection for 10 days starting immediately after surgery. The Pref-1-treated group showed lower blood glucose levels, as assessed using an IP glucose tolerance test (GTT), and decreased area under the curve of glucose values at 4 weeks after pancreatectomy (Fig. 5a,b). The regenerating capacity of the pancreas was improved in the Pref-1-treated group, as shown by the increase in Ki67-positive cells (Fig. 5c). In particular, more abundant small ductule formation (Fig. 5d) and enhanced pancreatic ductal (Fig. 5e) and β-cell (Fig. 5f) proliferation capacity were noticed in the Pref-1-treated group. In addition, the proportional area of pancreatic ductal and β-cells was increased by Pref-1 at 1 week after pancreatectomy. β-cell area was further increased at 4 weeks by Pref-1 compared to the control group (Fig. 5g). Increased expression of PDX1 and SCG2 and decreased expression of FOXO1 were also detected after Pref-1 treatment (Supplementary Figure 6), which was in line with *in vitro* data. Together, these data indicate that exogenous Pref-1 improves glucose homeostasis by accelerating pancreatic ductal and β-cell regeneration after injury.

## Discussion

Surrogates of the pancreatic β-cells from various sources or inducing the regeneration of the β-cells in the pancreas are promising options for the treatment of insulin-dependent diabetes mellitus because of the shortage of human islets for transplantation. Pancreatic duct cells have been considered as possible progenitor cells that have the potential to differentiate into insulin-positive cells^12^,^13^,^14^,^15^,^16^. Based on the known roles of Pref-1 and our previous observation that it is expressed in the duct cells of the embryonic pancreas and in the regenerating foci after a partial pancreatectomy^11^, we examined whether Pref-1 might induce ductal cell differentiation into β-like cells and promote insulin secretion.

**Figure 6:**
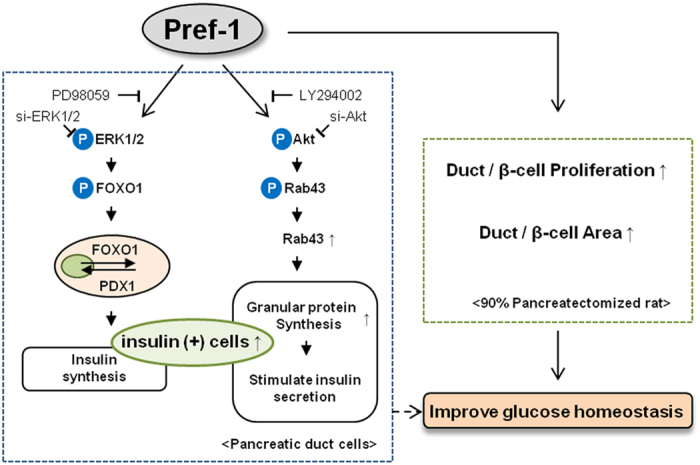
Schematic representation of the effects of Pref-1 in pancreatic ductal cells and pancreatectomized rats.

Our findings suggest that two independent pathways are involved in the induction of both insulin synthesis and secretion by Pref-1, improving glucose homeostasis (Fig. 6). First, Pref-1 activates PDX1 through the ERK-FOXO1 pathway and induces insulin synthesis. Pref-1 has previously been shown to promote the phosphorylation of ERK1/2 in a time-dependent manner in mouse embryonic fibroblasts^18^. Downstream of ERK, the FOXO1 plays important roles in pancreatic β-cell differentiation, neogenesis, proliferation, and stress resistance^27^. FOXO1 is directly phosphorylated by ERK, p38, and Akt in various cells and translocates from the nucleus to the cytoplasm^17^,^19^. In human umbilical vein endothelial cells, MEK/ERK and PI3K/Akt pathways are shown to synergistically regulate FOXO phosphorylation^19^. These evidences led us to hypothesise that Pref-1 promotes pancreatic duct cell proliferation and differentiation through the ERK, FOXO1, and Akt signaling pathway. However, by studying with a series of phosphorylation inhibitors, our results clearly show that nucleocytoplasmic translocation of FOXO1 and PDX1 is under the control of ERK but not Akt in pancreatic duct cells. Therefore, the effects of Pref-1 in inducing ductal cell differentiation into β-like cells and increased insulin synthesis were mediated by the ERK-FOXO1-PDX1 pathway.

Concurrently, Pref-1 induces Akt-Rab43 pathway, facilitating granular protein synthesis and GSIS. In searching the downstream signaling of Pref-1-induced Akt phosphorylation from the proteomics analysis data, Rab43 was considered as a strong candidate molecule. Rab proteins are Ras-related small GTPases which control vesicle movement and tethering membrane events in membrane trafficking. Among many Rab proteins, Rab43 has been identified to have a key role in the biogenesis and maintenance of a functional Golgi structure, and in the regulation of anterograde trafficking of cargo from endoplasmic reticulum to Golgi^24^,^28^. These functions of Rab43 may have relevance to the course of insulin processing and secretion. Furthermore, some Rab GTPase-activating proteins (GAPs) are known regulators of insulin signaling. For example, RacI, which is a member of the small GTPase family, regulates pancreatic islet morphogenesis^29^, and Rab-GAP AS160 is a downstream effector of PKB/Akt signaling in adipocytes, muscle, and pancreatic β-cells^30^,^31^. In this study, we first showed that Akt phosphorylation by Pref-1 treatment increases Rab43 synthesis and phosphorylation and that it stimulates the formation of intracellular vesicular granules, such as chromogranin and synaptophysin, within pancreatic duct cells. By inhibiting Akt in this context, we confirmed that these processes were indeed regulated by the Akt pathway. Interestingly, the expression of various vesicular proteins was altered in a time-dependent manner after Pref-1 overexpression. Synaptophysin expression increased transiently, and then disappeared over time. After the disappearance of synaptophysin in cells, we observed maintained expression of chromogranin A and SCG2, both of which are members of the granin family^32^,^33^. These observations might recapitulate the processes of pancreatic β-cell development from precursor cells and represent important steps for GSIS in mature β-cells.

During pancreatic development and regeneration after pancreatectomy, Pref-1 is not expressed in fully differentiated mature cells; however, it is detected in small ductules of regeneration foci after pancreatectomy (Supplementary Figure 1b). Moreover, in porcine neonate pancreas cell clusters, Pref-1 is coexpressed with synaptophysin and pancytokeratin (PanCK), which is expressed in pancreas duct cells (Supplementary Figure 1d,e). Therefore, we studied the effect of exogenous Pref-1 on the regenerating pancreas in a rodent model of diabetes by performing partial pancreatectomy. In concordance with the *in vitro* data, Pref-1 treatment enhanced pancreatic ductal and β-cell proliferation and regeneration, resulting in the improvement of glucose homeostasis. Taken together, a novel role of Pref-1 in the regulation of systemic glucose homeostasis was identified using pancreatectomised diabetic animal models.

Although Pref-1 is widely expressed in embryonic tissues and most cells of the pancreas, it becomes predominantly confined to a few specific cell types in adults^10^,^34^,^35^. In pancreas, Pref-1 expression becomes restricted to insulin producing β-cells during development. However, the role of Pref-1 in the β-cell remains unknown. A recent study examining the phenotype of pancreatic β-cell-specific Pref-1 knockout mice failed to demonstrate any effects shown in the global Pref-1 null mice. These mice were fully viable, showed no changes in growth and in the size, number or morphology of adult islets, suggesting that Pref-1 is not essential for the development of β-cells^35^. In contrast to the endocrine pancreas, our results indicate that Pref-1 might have an important role in the pancreatic ductal cells. To our knowledge, this is the first observation showing that Pref-1 induces differentiation of pancreatic ductal cells into insulin-producing β-like cells.

Because the Pref-1 receptor is yet unknown^36^, identification of the putative receptor would be important to further delineate the signaling pathways involved in the action of Pref-1. It also needs to be known whether the action of Pref-1 is regulated by cell non-autonomous manner or autocrine/paracrine effect. Detection of Pref-1 protein in culture medium from Pref-1-overexpressing cells supports the latter hypothesis (Supplementary Figure 2b), although further experiment needs to be performed. To appraise the possibility of clinical application, it would be interesting to find a link between the levels of Pref-1 and the metabolic status in human. It has been speculated that Pref-1 levels in early life might be associated with adipocyte numbers and the expandability of adipose tissue which could affect the vulnerability to the development of future diabetes^37^. Cross-sectional studies measuring serum levels of soluble Pref-1 in adults described that it is correlated with the degree of obesity, insulin sensitivity and diabetes status^38^,^39^,^40^. Of note, our recent study demonstrated that women with low serum Pref-1 concentrations have an increased risk of developing diabetes in the future suggesting a possible role of Pref-1 in the pathogenesis of type 2 diabetes^41^.

Some limitations of our experiment need to be acknowledged. First, because we used PANC1 cells which retain the properties of poorly differentiated tumor cells, they might differ from normal ductal differentiation state. Second, it is not clear whether Pref-1 induced the transdifferentiation of duct cells or acted as a de-differentiation factor allowing the proliferation of progenitor cells. Genetic lineage-tracing studies would be needed to answer this question. Third, it is possible that systemic administration of Pref-1 might have led to alterations in other tissues which may influence glucose homeostasis. Lastly, we were unable to address if signaling pathways similar to those characterized *in vitro* are also active in the regenerating pancreas *in vivo*.

In conclusion, Pref-1 activation induced the expression of the insulin mRNA and protein and increased intracellular insulin content and GSIS. Pref-1 led to pancreatic duct cell differentiation into β-like cells and increased insulin synthesis by activating ERK-FOXO1-PDX1 pathway. Granular protein synthesis and GSIS were controlled by the Akt-Rab43 pathway. Moreover, Pref-1 treatment triggered pancreas regeneration and improved glucose homeostasis *in vivo*. These findings open a new possibility of applying this molecule to the treatment of diabetes.

## Methods

### Animal Experiments

The Animal Care Committee of The Catholic University of Korea approved the experimental protocol, and all procedures performed followed the ethical guidelines for animal studies. Three-to-four-week-old Sprague Dawley rats weighing 50–60 g were anesthetized and underwent a 90% partial pancreatectomy^15^. Pancreatectomised diabetic rats were injected daily (IP) with 4 μg/kg of recombinant mouse Pref-1-Fc (provided by AdipoGen, Inc., Incheon, Korea) dissolved in phosphate-buffered saline (PBS), or with PBS as a control for 10 days after surgery. IPGTT was performed at 1 and 4 weeks after pancreatectomy. Glucose tolerance was assessed by IPGTT before and 1 and 4 weeks after pancreatectomy. After an overnight fast, 50% glucose solution was administered via IP injection (2 g/kg body weight). Blood samples were drawn from the tail vein at 0, 30, 60, 90 and 120 min after glucose administration. Glucose levels were measured using a glucometer (ARKRAY, Minneapolis, MN, USA). Pancreases were harvested for histological examination.

### Cell Culture and Transfection

The human epithelioid carcinoma cell line PANC1 used in this study was purchased from the American Type Culture Collection (Manassas, VA, USA). Cells were maintained in high-glucose Dulbecco’s Modified Eagle Medium supplemented with 0.44 M sodium bicarbonate, 0.02 M HEPES (pH 7.33), 10% (vol/vol) FBS, and 100 μg/mL antibiotic/antimycotic (GIBCO, Carlsbad, CA, USA). The rat insulinoma cell line INS-1 was maintained in RPMI1640 medium supplemented with 11.1 mM glucose, 10 mM HEPES, 2 mM L-glutamine, 1 mM sodium pyruvate, 50 M β-mercaptoethanol, 10% FBS, and 100 μg/mL antibiotic/antimycotic. Purified secreted human Pref-1-hFc fusion protein (AdipoGen, Inc.) was diluted to a 50 μg/mL working solution and delivered to cells at a final concentration of 50 ng/mL. The inhibitors were dissolved in DMSO to a stock concentration of 25 mM (PD98059) and 20 mM (LY294002; Cell Signaling Technology, Danvers, MA, USA). Cells were serum-starved 4 h before exposure. Transfection was carried out using Lipofectamine 2000 (Invitrogen, Carlsbad, CA, USA) according to the manufacturer’s instructions. To generate the Pref-1-overexpressing cell lines, we purchased the human Pref-1-expressing vector (pSPORT6-hDLK1) from Open Biosystems (Huntsville, AL, USA).

### siRNA transfection in PANC1 Cells

After a 2-day culture, PANC1 cells were reseeded into six-well plates at 2 × 10^5^ cells/well. After 24 h, the Rab43 siRNA (50 pmol, Santa Cruz Biotechnology Inc., Dallas, TX, USA), p44/42 MAPK (ERK1/2) siRNA (100 nM) and Akt siRNA (100 nM, Cell Signaling Technology) were delivered to cells via a lipid-mediated Lipofectamine 2000 Transfection Reagent. A nonrelated scramble siRNA (Invitrogen) and SignalSilence control siRNA (Cell Signaling Technology) were used as a transfection control.

### Cellular Fractionation

Cells were resuspended in 200 μL of buffer A (25 mM Tris, pH 8.0, containing 2 mM MgCl_2_, 0.5 mM dithiothreitol, and 0.01% PMSF [Sigma Chemical Co., St. Louis, MO, USA]) and incubated for 5 min. After the addition of 1.25 μL of 10% Nonidet P-40, the cells were incubated for 2 min. The homogenate was centrifuged for 20 min at 1,700 rpm, and the supernatant was designated the cytoplasmic protein fraction. The pellet was resuspended in 80 μL of buffer B (0.4 M LiCl, 10 mM Tris, pH 8.0, 0.5 mM dithiothreitol, and 0.01% PMSF) and incubated for 5 min. The homogenate was centrifuged at 12,000 rpm for 10 min, and the supernatant was considered as the nuclear protein fraction.

### Immunoprecipitation and Western Blot Analysis

Cells were lysed in PBS containing 1 mM Na_3_VO_4_, 1 mM NaF, 150 mM NaCl, 1% Nonidet P-40, 0.5% sodium deoxycholate, 0.1% SDS, 50 mM Tris-HCl (pH 8.0), and 1 mM PMSF. Cell lysates were precleared by the addition of protein G sepharose beads (Millipore, Billerica, MA, USA) and immunoprecipitated with protein G beads precoupled with an anti-Rab43 antibody (1:1,000, ab58030; Abcam, Cambridge, MA, USA). Immunoprecipitates or whole-cell lysates were fractionated by SDS-PAGE and transferred onto an Immobilon polyvinylidene difluoride membrane (Millipore, Billerica, MA, USA). After blocking, the membranes were incubated with one of the following antibodies: anti-DLK1 (1:1,000, sc25437), anti-SCG2 (1:1,000, sc50290), anti-p-Ser (1:500, sc81514; Santa Cruz Biotechnology Inc.), anti-phosphorylated Akt (1:1000, #9271), anti-phosphorylated ERK1/2 (1:1,000, #9101), anti-ERK1/2 (1:1,000, #9107), anti-phosphorylated FOXO1 (1:1,1000, #9461), anti-FOXO1 (1:500, #2880; Cell Signaling Technology, Danvers, MA, USA), anti-PDX1 (1:1000, ab3503; Chemicon, Temecula, CA, USA), anti-synaptophysin (1:500, M0776; Dako Cytomation, Glostrup, Denmark), Lamin B1 (1:1000, 33–2000; Thermo Scientific, Rockford, IL, USA), or anti-beta-actin (1:5,000, A5441; Sigma Chemical Co.). Proteins were visualized using an enhanced chemiluminescence kit in accordance with the manufacturer’s recommendations.

### RNA Extraction and RT-PCR

Total RNA was extracted from cells using the TRIzol reagent (GIBCO). Total RNA (1 μg) was used in the RT reaction with SuperScript^TM^ II (Invitrogen), according to the manufacturer’s instructions. PCR amplification was performed using recombinant *Taq* DNA polymerase, 10× buffer, and dNTP Mixture for PCR from a *TaKaRa Taq*^TM^ kit (TaKaRa Biomedical, Mountain View, CA, USA). All PCR primer sequences and PCR conditions are described in Supplementary Table 2.

### Insulin Content and Insulin Secretion

Cells were seeded in six-well culture dishes and grown in complete medium. The medium and cell lysates were harvested at the indicated time points. The incubation medium was gently centrifuged to remove floating cells. The cell density was determined after trypsinization in a Neubauer counting chamber. For the measurement of cellular insulin content, the cells were homogenized in acetic acid. Insulin content and secretion levels were measured using a commercially available RIA kit (Millipore) according to the manufacturer’s protocol.

### GSIS

Cultured cells were washed in Krebs-Ringer-bicarbonate (KRB) washing buffer (130 mM NaCl, 3.6 mM KCl, 1.5 mM CaCl_2_, 0.5 mM MgSO_4_, 0.5 mM KH_2_PO_4_, 2.0 mM NaHCO_3_, and 10 mM HEPES) and incubated in KRB buffer containing 1 mM glucose for 1 h. The cells were then stimulated for 1 h in KRB buffer containing 25 mM glucose. C-peptide and insulin concentrations were measured with a RIA kit (Millipore).

### Immunochemistry

Pancreatic tissues were fixed with 4% paraformaldehyde (Sigma Chemical Co.) at 4 °C for 16 h, and processed using standard paraffin-embedding protocol and sectioned at a 4 μm thickness. For the immunostaining of cultured cells, cells were fixed with 4% paraformaldehyde for 15 min. Before staining, cells were permeabilized with 0.5% triton X-100 for 20 min. Nonspecific binding was blocked by incubation for 20 min at room temperature with 1.5% of the serum from the species in which the secondary antibody was raised. Anti-mouse and anti-rabbit biotinylated antibodies and normal serum were purchased from Vector Inc (Tokyo, Japan). Cells were incubated at 4 °C overnight with one of the following antibodies: anti-DLK1 (1:100), anti-PDX1 (1:200), anti-FOXO1 (1:100), anti-synaptophysin (1:50), anti-SCG2 (1:100), anti-Ki67 (1:200, ab15580, abcam), anti-PanCK (1:100, 18–0059) or anti-insulin (1:100, 18–0059, Invitrogen). The secondary antibodies were applied for 1 h at room temperature, and the samples were then mounted with an anti-fade medium that consisted of 90% glycerol in PBS (pH 8.6) including 0.1 M DABCO (Sigma Chemical Co.). For immunofluorescence, Texas Red-conjugated affiniPure donkey anti-guinea pig IgG, FITC-conjugated affiniPure donkey anti-rabbit IgG, and streptavidin-conjugated FITC and Texas Red (Jackson Immuno-Research Lab, West Grove, PA, USA.) were used as secondary antibodies.

### Image Acquisition

Digital images at two or three fluorescent emission wavelengths were acquired using a Bio-Rad confocal microscope. The light sources were an Argon/He and multiphoton laser with excitation wavelengths of 368–647 nm. FITC and Texas Red were excited at 488 nm and 568 nm, respectively. FITC and Texas Red images were scanned separately and captured as overlapped images.

### Morphometric analysis

For β-cell and ductal cell proliferation assays, sections were double stained for Ki67 and insulin or PanCK, respectively. All islets or ducts were imaged using Leica Application Suite 4.0 (Leica Microsystems, Wetzlar, Germany). Total β-cell or ductal cell number was assessed by counting nuclei surrounded by cytoplasmic insulin or PanCK immunostaining, and the proliferating cells were assessed by counting nuclei stained with Ki67 within cytoplasmic insulin or duct immunostaining. Cell proliferation ratio was calculated by dividing Ki67-positive cell number by total insulin-positive or PanCK-positive cell number. To quantify fractional β-cell or ductal cell area, the total pancreas, insulin-positive, and PanCK-positive areas were selected using Adobe Photoshop for each image. These were measured as the ratio of the insulin-positive or PanCK-positive area over the total tissue area of the entire section.

### Statistical Analysis

Data are presented as means ± SE. Unpaired Student’s t-test or ANOVA were performed as appropriate. A *P* value <0.05 was considered statistically significant.

## Additional Information

**How to cite this article**: Rhee, M. *et al.* Preadipocyte factor 1 induces pancreatic ductal cell differentiation into insulin-producing cells. *Sci. Rep.* **6**, 23960; doi: 10.1038/srep23960 (2016).

## Supplementary Material

Supplementary Information
