# Supplementary material for: Preadipocyte factor 1 induces pancreatic ductal cell differentiation into insulin-producing cells
Source: Sci Rep. 2016 Apr 5;6:23960. doi: 10.1038/srep23960 (PMC4820710; doi:10.1038/srep23960)
Supplement: Supplementary Information [file srep23960-s1.pdf]

Supplementary Information

Preadipocyte factor 1 induces pancreatic ductal cell differentiation into insulin-producing cells

Marie Rhee, Seung-Hwan Lee, Ji-Won Kim, Dong-Sik Ham, Heon-Seok Park, Hae Kyung Yang, Ju-Young Shin, Jae-Hyoung Cho, Young-Bum Kim, Byung-Soo Youn, Hei-Sook Sul, Kun-Ho Yoon

Table S1. List of up- and down-regulated proteins induced by Pref-1 in PANC1 cells.

Spot.	Accession Number	Protein name	Reported Function
**Up-regulation**			
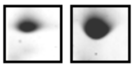	gi|50234889	**RAB43 protein**	**- Belongs to the small GTPase superfamily. Rab family**<br>**- ER-Golgi trafficking, GTP binding**<br>**- Biogenesis and maintenance of a functional Golgi structure**
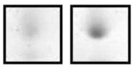	gi|34303941	zinc finger protein 567	- May be involved in transcriptional regulation<br>- DNA binding, Zinc ion binding
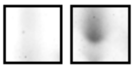	gi|110815807	zinc finger protein 169	- May be involved in transcriptional regulation<br>- Highly expressed in kidney, weakly expressed in heart, liver, spleen, and small intestine. Not expressed in adult brain or spinal cord
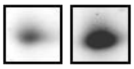	gi|39939749	immunoglobulin heavy chain variable region	
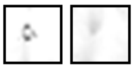	gi|119607456	zinc finger homeodomain 4, isoform CRA_b	- May play a role in neural and muscle differentiation<br>- May be involved in transcriptional regulation
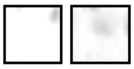	gi|42794775	thioredoxin domain containing 5 isoform 2	**thioredoxin family*; antihypoxia-induced apoptosis, cell proliferation, differentiation, and angiogenesis<br>- essential protective effect against hypoxia-induced cell apoptosis in Ecs
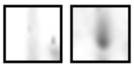	gi|119594432	eukaryotic translation elongation factor 1 gamma, isoform CRA_d	- Probably plays a role in anchoring the complex to other cellular components<br>- Calcium ion binding, phospholipid binding, transcription coactivator activity, translation elongation factor activity
**Down-regulation**			
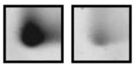	gi|194374891	unnamed protein product	
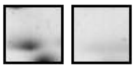	gi|71042776	Chain A, 14-3-3 Protein Theta (Human) Complexed To Peptide	
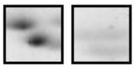	gi|164691151	unnamed protein product	

Table S2. PCR primer sequences and their product size.

Primer		Sequences (5' - 3')	Product Size	Annealing (℃)
Pref-1	Sense	GGATTCTGCGAGGATGAC	170 bp	57
	Antisense	GCCCGAACATCTCTATCAC		
FOXO1	Sense	AACCAGCTCAAATGCTAGTACCATC	178 bp	52
	Antisense	AAGGTTCTCCATGTTTTTCTG		
PDX1	Sense	CCCATGGATGAAGTCTACC	225 bp	57
	Antisense	GTCCTCCTCCTTTTTCCAC		
Insulin	Sense	AACCAACACCTGTGCGGCTC	320 bp	52
	Antisense	AAGGGCTTTATTCCATCTCTCTCG		
Rab43	Sense	CCTTCACAGGGATGGTAGCTG	148 bp	55
	Antisense	GGAGCCTTTGAAACATCCTG		
Synaptophysin	Sense	TGTACTTTGATGCACCCACCT	425 bp	54
	Antisense	CAGCCTGTCTCCTTAAACACG		
SCG2	Sense	CCCTACCAAGGTGTCTCTGT	149 bp	53
	Antisense	GGTGCAGACTGAGGCTCATTT		
β-Actin	Sense	ATCATGTTTGAGACCTTCAACACCC	552 bp	55
	Antisense	CATGGTGGTGCCGCCAGACAG		

Figure S1. Pref-1 expression of the embryonal and regenerative rat pancreas and monolayer-cultured porcine neonatal pancreas cells (NPCCs). (**a**) Pref-1 shown in the rat embryonal pancreas at E20. Pref-1 expression was restricted to only the small ductules (red arrow). Scale bar, 100 µm. (**b**) Expression of Pref-1 in partial pancreatectomized rat pancreas. After pancreatectomy, Pref-1 was strongly expressed in the small regenerative duct cells at foci of regeneration and then completely disappeared at day 7 after pancreatectomy. Scale bar, 100 µm. (**c**) NPCCs that consist mainly of duct cells and protodifferentiated cells were stained with Pref-1 and were then analyzed by fluorescence-activated cell sorting (FACS). Isolated Pref-1 -/+ cells were harvested, total RNA was isolated and RT-PCR analysis for protodifferentiated cell markers was performed. Pref-1-expressed pancreatic cells co-express with PDX1 and synaptophysin (SYP). (**d**) Co-expression of Pref-1 and SYP were also observed in NPCCs (left panel) and foci in the regenerating pancreas of 90% pancreatectomized rat (right panel) by immunocytochemistry. Scale bar, 50 µm. (**e**) Co-expression of Pref-1 and pancytokeratin (PanCK) was observed in NPCCs. Scale bar, 50 µm.

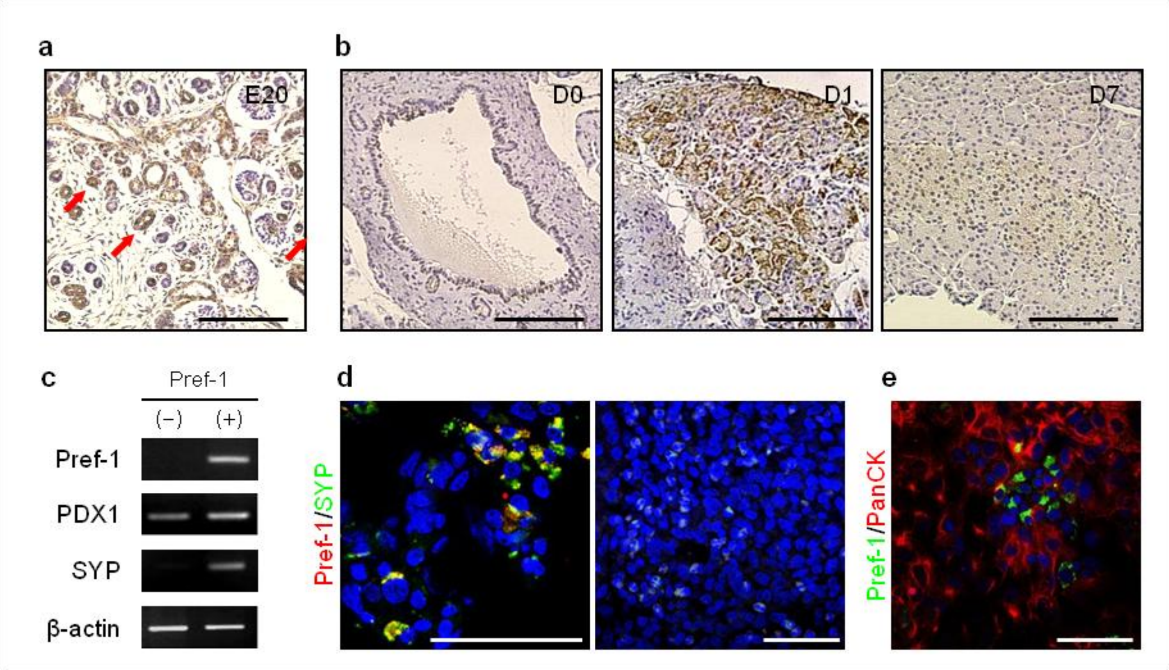

Figure S2. Transduction of Pref-1 in PANC1 cells. (**a**) Pref-1 promotes the phosphorylation of ERK1/2, Akt and FOXO1 by transduction of pSPORT6-hDLK1 as Pref-1. Cells were harvested at 48 hr after transfection of pCMV or pSPORT6-hDLK1. (**b**) Secretion of Pref-1 protein. Cells were transfected with pCMV or pSPORT6-hDLK1 for the indicated times. Pref-1 protein was detected by western blot assay in the immunoprecipitates from corresponding cell culture media (supernatant). Immunoprecipitation and western blot assays were performed using an antibody directed against DLK1. (**c**) mRNA and protein expression and immunostaining of Pref-1 at day 2 after transfection with either pCMV or pSPORT6-hDLK1.

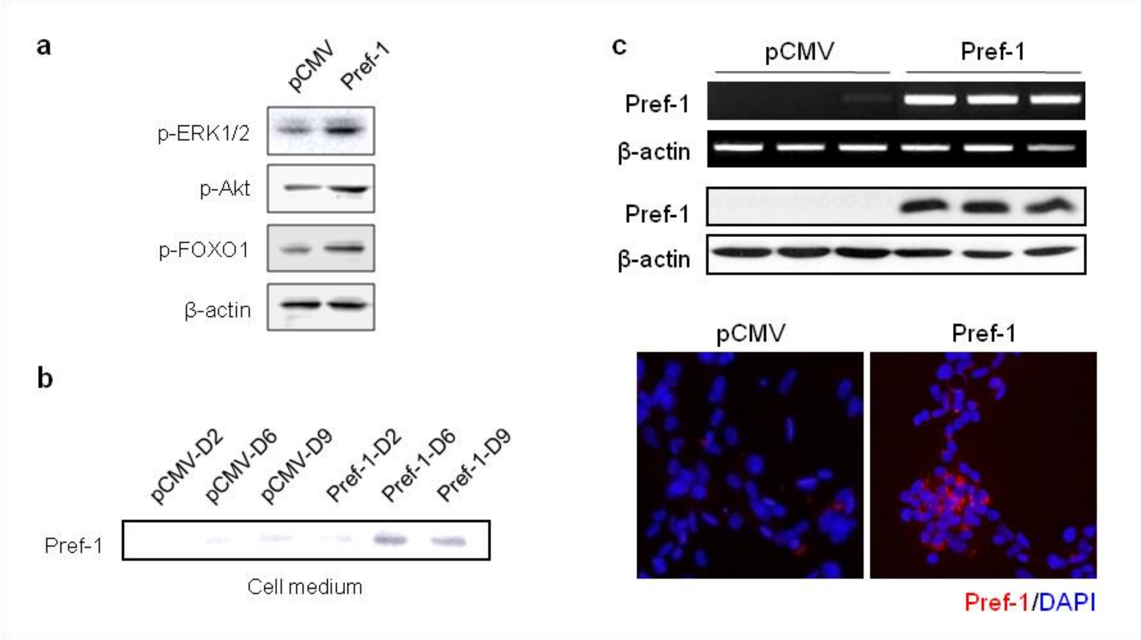

Figure S3. Identification of Rab43 by 2DE gel electrophoresis and mass spectrometry analysis.

Proteins with increased or decreased expression in Pref-1-treated cells compared with control were identified by 2DE gel electrophoresis. 10 protein spots up-/down-regulated in Pref-1 treated cells were selected for matrix-assisted laser desorption/ionization time-of-flight mass spectrometry (MALDI-TOF MS) analysis. High magnification views of the regions of the gels framed (arrowhead) are shown in Table S1.

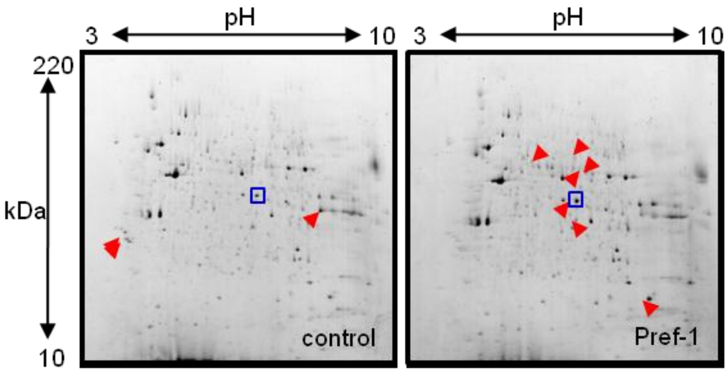

Figure S4. The effect of Pref-1 on C-peptide synthesis and glucose-stimulated C-peptide secretion in PANC1 cells. (**a**) Cellular C-peptide content in Pref-1-overexpressing cells at the indicated time. Cells were harvested at day 9 after transfection with pCMV (white bar) or Pref-1 (solid bar). n = 3, $^{**}P < 0.01$ (means ± SE). (**b**) Glucose-stimulated C-peptide secretion into the culture medium in Pref-1-overexpressing cells. Cells were harvested at day 9 after transfection with pCMV (white bar) or Pref-1 (solid bar). n = 3, $^{*}P < 0.05$, $^{**}P < 0.01$ (means ± SE).

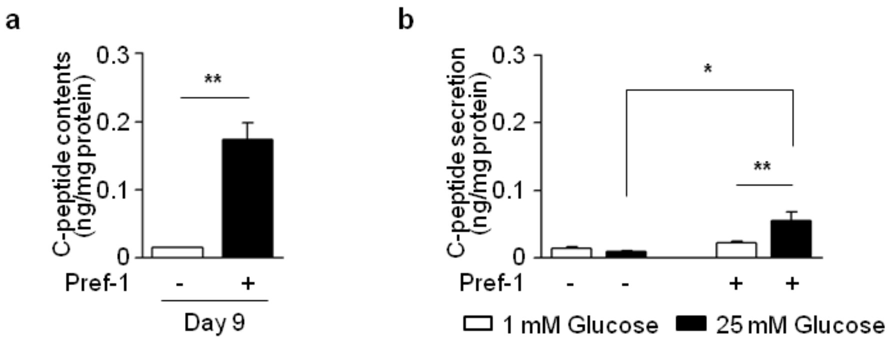

Figure S5. The effect of Pref-1 on insulin contents and glucose-stimulated insulin secretion in rat islets. Insulin contents (**a**) and glucose-stimulated insulin secretion (**b**) in rat islets after transfection with pCMV (white bar) or Pref-1 (solid bar). Isolated rat islets were treated with pCMV or Pref-1 for 3 days (**c**). n = 3. (means ± SE).

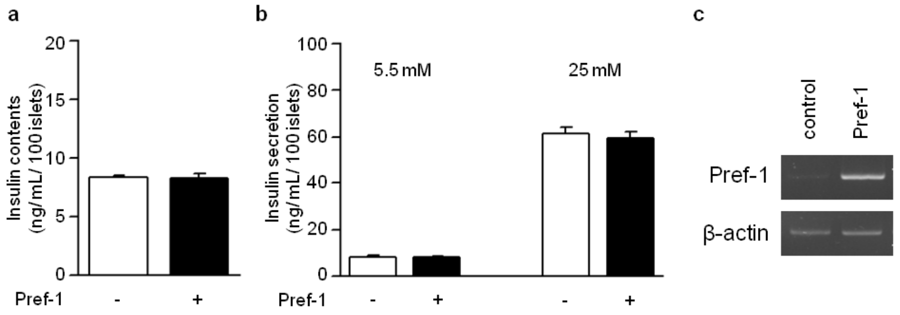

Figure S6. Immunostaining of PDX1, FOXO1, and secretogranin (SCG) 2 in pancreatectomized rats. All tissues were harvested at 1 week after pancreatectomy. Left panel shows islets and right panel shows duct-enriched areas. Scale bar, 50 μm.

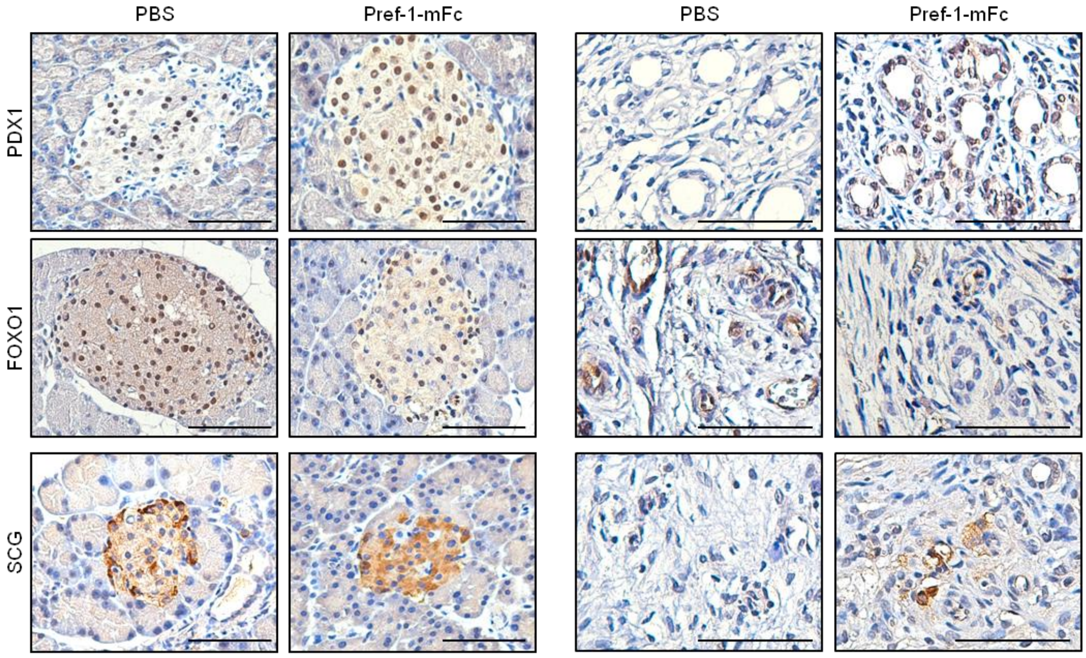

Supplementary Methods

Animal Experiments. Pregnant female rats (Sprague Dawley rats) were anesthetized and killed by an overdose of the anesthetics. Embryos were recovered from pregnant rats at E20. All pancreatic tissues were fixed with 4% paraformaldehyde at 4℃, and processed using standard paraffin-embedding protocol and sectioned at a 4 μm thickness.

Preparation of porcine NPCCs. Porcine NPCCs were isolated from 1-2-days-old neonatal pigs as previously described[1]. To culture NPCC cells in a monolayer, the cell clusters were broken up by gentle aspiration after 1 day of free-floating in culture, and were cultured in DMEM supplemented with 10% fetal bovine serum in a humidified chamber containing 5% CO2 for 3 days. For Pref-1 staining, cells were washed and resuspended with PBS containing 1% FBS, then incubated with anti-DLK1 antibody (1:100 in PBS containing 1% FBS) for 1 hr at 4℃. Sorting and analysis were carried out on a FACSVantage SE Flow cytometer (Becton-Dickinson, San Jose, CA).

Rat Islet Isolation and Culture. Rat pancreatic islets were isolated from Sprague Dawley rats (200–230 g) by digesting the pancreatic duct with collagenase P (1 mg/ml in Medium 199). After digestion, the islets were separated with Histopaque-1077 (Sigma Chemical Co., St. Louis, MO, USA). The islets were cultured in RPMI 1640 medium containing 10% FBS, and 100 μg/mL antibiotic/antimycotic (GIBCO). Cultured cells were washed in Krebs-Ringer-bicarbonate (KRB) washing buffer and incubated in KRB buffer containing 5.5 mM glucose for 1 h. The cells were then stimulated for 1 h in KRB buffer containing 25 mM glucose. Insulin concentrations were measured with a RIA kit (Millipore).

2 DE Gel electrophoresis and MALDI-TOF Mass Spectrometry analysis. Cells were sonicated with sample lysis buffer (7M urea, 2M thiourea, 65 mM 3-[(3-cholamidopropyl) dimethylammonio]-1-propanesulfonate (CHAPS), 100 mM dithiothreitol, 2% Pharmalyte (pH 3–10), complete protease inhibitor (Roche, Basel, Switzerland)), and DNase I (10 U/L mg proteins; Boehringer-Mannheim, Mannheim, Germany) was then added. Samples (1 mg/ml) were applied to strips by in-gel rehydration. Isoelectric focusing was performed at 20℃ with a current limit of 50 mA/strip as follows: Immobiline DryStrips (18 cm, pH 3–10; Pharmacia, Uppsala, Sweden) were equilibrated in 375 mM Tris-HCl (pH 8.8), 6 M urea, 20% glycerol, 2% SDS, and 0.01% bromophenol blue with 10

mM tributyl phosphine. The strips were loaded on the top of 9%–16% vertical SDS gradient slab gels for the second dimension separation. Protein spots were stained with Coomassie Brilliant Blue G-250 (Bio-Rad Laboratories, Richmond, CA, USA). Images were obtained with a GS-710 image scanner (Bio-Rad Laboratories) and the 2-DE protein patterns were processed with Melanie 5 software (Gene-Bio, Geneva, Switzerland). Spots of interest were excised with end-removed pipette tips to accommodate various spot diameters. The gel slices were destained, dehydrated with 50 ml of ammonium bicarbonate/acetonitrile (60:40) at room temperature, dehydrated with 50 ml 100% acetonitrile solution, and then dried with a Speedvac. The dried gels were digested with trypsin solution, and the tryptic peptides were loaded onto columns using GELoader tips (Eppendorf, Hamburg, Germany). After equilibration with 0.1% TFA, the peptides were eluted in 70% acetonitrile/0.1% TFA solution and dropped onto a MALDI plate (Applied Biosystems, Foster City, CA, USA). MALDI-TOF mass spectrometry was performed with an Applied Biosystems Voyager DE-PRO spectrometer. The instrument was operated with an accelerating voltage of 20 kV, positive ion reflection mode, 74.5% voltage grid, 0% guide wire voltage, and delay time of 75 ns. The spectra were internally calibrated using the trypsin autolysis products (842.51 and 2211.11 Da), and monoisotopic peptide masses were assigned and used for database searches. Proteins were identified by searching the SWISS-PROT and NCBI databases using MSFit (Protein Prospector; http//www.prospector.ucsf.edu) and MASCOT (Matrix Science; http//www.matrixscience.com). All searches were analyzed with a 100 ppm mass tolerance.

Supplementary Reference

1. Korbutt, G. S., Elliott, J. F., Ao, Z., Smith, D. K., Warnock, G. L. & Rajotte. R. V. Large scale isolation, growth, and function of porcine neonatal islet cells. *The Journal of clinical investigation* **97**, 2119-2129 (1996)
